# Supplementary material for: A computational offloading optimization scheme based on deep reinforcement learning in perceptual network
Source: PLoS One. 2023 Feb 24;18(2):e0280468. doi: 10.1371/journal.pone.0280468 (PMC9955616; doi:10.1371/journal.pone.0280468)
Supplement: Attachment — Submitted filename: Authors_Response.pdf [file pone.0280468.s001.pdf]

A computational offloading optimization scheme based on deep reinforcement learning in perceptual network

Authors' Response

We are pleased to submit a revised version of our manuscript. First, we would like to thank the editors and reviewers for their valuable time reviewing our manuscript and for their valuable comments. In response to the reviewers' comments, we have made revisions one by one. Below is our response to the review comments. Additionally, we thank the editor again for allowing us to revise and resubmit our manuscript.

Thank you once again for your valuable time, insightful suggestion as well as positive comments. We hope the above-detailed response has clarified the essential issues, and our revised manuscript will meet your approval.

Sincerely:

The Authors

Response to Reviewer 1st Comments

This paper presents a nice idea on using deep reinforcement learning to investigate a computation offloading optimization scheme for the perception layer. I think it is an interesting approach, have enough novelty, and the paper is on the whole quite well written. The issues to be addressed are:

Comment # 1: Please clarify the difference between your proposed algorithm and the idea of reference [10].

Response: Thank you for your suggestion and comment. Reference [10] is to use A3C to solve the computational task offloading model. Both A3C and DDPG are improved actor-critic algorithms, but DDPG has a small action space variance and will converge more easily. This paper mainly refers to the model construction of the literature [10]. It uses the DDPG algorithm to adaptively adjust the computing task offloading strategy of the IoT terminal to improve the offloading efficiency and reduce the task delay. In addition, we also conduct a comparative analysis of A3C and DDPG algorithms in the experimental part. A3C and DDPG perform very close in every scene, but overall, DDPG performs slightly better than A3C. Reference [10] in the revised manuscript at location [12].

Comment # 2: Show the impact of data block size and block interval (from parameter settings) on average delay.

Response: Thank you for your comment. This paper mainly focuses on optimizing the problem of local computing or offloading the tasks from the IoT terminal to the edge server for computing. The parameter setting in the experiment is the data size of the unloading task. The set data size is within the acceptable calculation range of the mobile edge server. When the task is offloaded, it can adaptively find a suitable edge server for calculation. It won't affect the average delay too much. In addition, the default size of the data block is generally 64MB or 128MB. The revised text is highlighted in RED. For your convenience, the revised text is given as follows.

In addition, s^t represents the data size of the offloaded task. The set data size is within the acceptable calculation range of the mobile edge server. When the task is offloaded, it can adaptively find a suitable edge server for calculation. It won't affect the average delay too much.

Comment # 3: In addition to the average delay, show the impact of the parameters (at least one of them) on the throughput.

Response: Thank you for your comment. This paper mainly models and conducts experiments for minimizing the average delay and maximizing the task completion rate and is not designed for throughput. In the reward setting, if the task is completed before the deadline, that is, if the task completion rate is relatively high, a high reward will be set. Otherwise, a relatively low reward will be set. During the optimization process, the solution will converge in the direction of high reward to ensure a high task completion rate and low average delay. In the future, we will also consider more objectives for experiments to maximize performance.

Response to Reviewer 2nd Comments

This paper investigates a computation offloading optimization scheme for the perception layer. The algorithm can adaptively adjust the computational task offloading policy of IoT terminals according to the network changes in the perception layer. Although the work has potential, several major concerns need to be addressed before the paper can be accepted for publication:

Comment # 1: The optimization problem in Section 3.1.3 is not clear. Specifically, it is not clear from Equation (6) what the authors are trying to optimize. How can the task completion rate be optimized by maximizing the local and service execution times?

Response: Thank you for your comment. Equation (6) is used to calculate the task completion time. Both task delay and task completion rate are calculated based on Equation (6). In the reward setting, if the task is completed before the deadline, that is, if the task completion rate is relatively high, a high reward will be set. Otherwise, a relatively low reward will be set. During the optimization process, the solution will converge in the direction of high reward to ensure the task completion rate. The revised text is highlighted in RED. For your convenience, the revised text is given as follows.

Our optimization goal considers the expected task delay and the task completion rate (TCR). According to the above formula, the task completion time is:

$$t_i = \max(\mathrm{t_i^{local}}, (\mathrm{t_i^{cal+que}} + \mathrm{t_i^{trans}})) \tag{1}$$

Assuming that there are P tasks in total, the expected delay of all tasks (which can also be understood as the average task completion time of P tasks) can be expressed as:

$$E_t = \frac{\sum_{i=1}^{P} t_i}{P} \tag{2}$$

Assuming that there are C tasks completed within the deadline, the task completion rate of all tasks can be expressed as:

$$TCR = \frac{C}{P} \times 100\% \tag{3}$$

This paper aims to maximize the TCR while making the task waiting time as small as possible. Therefore,

when the task is not completed within the deadline, a negative reward is given, and when the task is completed within the deadline, a high positive reward is given. We set the reward as:

$$R_i = \begin{cases} -t_i & t_i > t_{d_i} \\ log_{0.995}(1 - \frac{1}{e^{\sqrt{t_i}}}) & t_i < t_{d_i} \end{cases} \tag{4}$$

Where t_{d_i} represents the task deadline.

Comment # 2: Moreover, no constraints are defined for the optimization problem, which makes it incomplete.

Response: Thank you for your comment. We added constraints to the problem in model building to make the article more complete. The revised text is highlighted in RED. For your convenience, the revised text is given as follows.

In addition, we need to consider some constraints when calculating. n represents the total number of tasks, and M represents the total number of MECs. i represents the task index, and j represents the MEC index. M_i denotes the MEC-available set of task n_i, and M_{ij} denotes that task n_i is executed on MEC M_j. t_i represents the task completion time of task n_i. B_{ij} represents the task execution time of task n_i on MEC M_j.

$$X_{ij} = \begin{cases} 1, & if\ task\ n_i\ is\ executed\ on\ MEC\ M_j \\ 0, & others \end{cases} \tag{5}$$

$$\sum_{j \in M_j} X_{ij} = 1, \forall i, j \tag{6}$$

$$B_{ij} \leq t_i \tag{7}$$

$$B_{ij} \geq 0, t_i \geq 0 \tag{8}$$

Among them, constraint (6) indicates that one MEC can be selected from the set of optional MECs for each task n_i for execution, but each task can only be executed on one MEC. Constraint (7) restricts that the task execution time of each task n_i on MEC M_j cannot exceed its task completion time. constraint (8) qualifies the

non-negativity of all parameters.

Comment # 3: It is not clear what novel contributions the authors bring by employing Deep Reinforcement Learning (DRL) for offloading. Is the internal structure of DRL tuned for the considered problem, or is it just used as a blackbox approach?

Response: Thank you very much for your suggestion. This paper builds an optimization model for offloading computing tasks of IoT terminals under the perception layer network and uses deep reinforcement learning to adapt the computing task offloading strategy of IoT terminals. This paper does not adjust the internals of DDPG but uses it as a blackbox approach. But in future research, we will also consider further optimization of DDPG to make the unloading more efficient.

Comment # 4: Important literature on offloading/scheduling is missing such as:

- Ad hoc vehicular fog enabling cooperative low-latency intrusion detection. IEEE Internet of Things Journal, 8(2), 829-843 (2020).

- Intelligent task prediction and computation offloading based on mobile-edge cloud computing. Future Generation Computer Systems, 102, 925-931 (2020).

- Multi-user multi-task computation offloading in green mobile edge cloud computing. IEEE Transactions on Services Computing, 12(5), 726-738 (2018).

- A trust and energy-aware double deep reinforcement learning scheduling strategy for federated learning on IoT devices. In International Conference on Service-Oriented Computing (pp. 319-333) (2020).

Response: Thank you for your comment. We carefully read several of the important references you suggested and added them to the appropriate place in the paper to make it more persuasive. For your convenience, We added literature to the paper as follows.

The Internet of Things (IoT) has significant advantages over traditional communication technologies. However, IoT devices have limited resources [1].

Generally speaking, limited resources are solved by shifting the computational workload to other devices with better resources and offloading computation [2, 3].

On the other hand, the extensive distribution of edge computing nodes and the powerful offloading capability [4] greatly facilitate the task computing and data transmission of IoTs terminals.

Comment # 5: What is the computational complexity of Algorithm 1? Can it be used to make real-time offloading decisions?

Response: Thank you for your comment. We have added an analysis of the time complexity of Algorithm 1. In addition, the DDPG algorithm can adapt to dynamic environments. It can judge whether the task is offloaded or not based on factors such as real-time network conditions and the operating conditions of the mobile device itself. DDPG algorithms can be used to make real-time decisions. The revised text is highlighted in RED. For your convenience, the revised text is given as follows.

From the pseudo-code of Algorithm 1, it can be seen that the time complexity of this algorithm is related to the size of the episode and the size of the time step in each episode. Line 2 takes an average of t_{11} time to run once, and runs N times repeatedly, so the running time is $t_{11}N$. Assume that running 4 to 13 rows in each time step takes an average of t_{12} time. Running T times consumes an average of $t_{12}T$ time and then repeats the operation N times. So the running time is $t_{12}TN$. So the total time to run is $t_{11}N + t_{12}TN$. When the values of N and T are relatively large, the coefficients of $t_{11}N$ and $t_{12}TN$ are negligible, so the time complexity is $O(N + NT)$. Because NT grows much faster than N, the time complexity of this algorithm is $O(NT)$.

Comment # 6: The environment within which the simulations have been done needs to be further clarified, i.e., programming language, framework, resource specifications, etc.

Response: Thank you for your suggestion. We further clarify the environment in which the simulation is performed, including programming languages, frameworks, resource specifications, etc., in the experimental section to complete the paper. The revised text is highlighted in RED. For your convenience, the revised text is given as follows.

The experiments in this paper are all implemented based on Python 3.8 programming under the Windows 10

system, and the DDPG algorithm is implemented based on the TensorFlow framework programming. The MEC server is equipped with an Intel Core i7-7700 CPU. In the standard configuration, 30 IoT devices are connected in each case.

Thank you once again for your valuable time, insightful suggestion as well as positive comments. We hope the above-detailed response has clarified the essential issues, and our revised manuscript will meet your approval again.

Looking forward to further advice you may have for us.

Sincerely:

The Authors

References

[1] A. Mourad, H. Tout, O. A. Wahab, H. Otrok, and T. Dbouk, "Ad hoc vehicular fog enabling cooperative low-latency intrusion detection," *IEEE Internet of Things Journal*, vol. 8, no. 2, pp. 829–843, 2020.

[2] Y. Miao, G. Wu, M. Li, A. Ghoneim, M. Al-Rakhami, and M. S. Hossain, "Intelligent task prediction and computation offloading based on mobile-edge cloud computing," *Future Generation Computer Systems*, vol. 102, pp. 925–931, 2020.

[3] W. Chen, D. Wang, and K. Li, "Multi-user multi-task computation offloading in green mobile edge cloud computing," *IEEE Transactions on Services Computing*, vol. 12, no. 5, pp. 726–738, 2018.

[4] G. Rjoub, O. Abdel Wahab, J. Bentahar, and A. Bataineh, "A trust and energy-aware double deep reinforcement learning scheduling strategy for federated learning on IoT devices," in *International Conference on Service-Oriented Computing*. Springer, 2020, pp. 319–333.
